# Decision-Aware Multi-Horizon Fault Prediction for Photovoltaic Inverters: Analysis of Threshold-Based Alarm Policies Under Operational Constraints

**DOI:** 10.3390/s26082463

**Published:** 2026-04-16

**Authors:** Jisung Kim, Tae-Yun Kim, Hong-Sic Yun, Seung-Jun Lee

**Affiliations:** 1 School of Geography, University of Leeds, Leeds LS2 9JT, UK; gyjki@leeds.ac.uk; 2 Department of Civil and Environmental Engineering, Sungkyunkwan University, Suwon 16419, Republic of Korea

**Keywords:** photovoltaic inverter, fault prediction, multi-horizon forecasting, predictive maintenance, time-series transformer, alarm policy

## Abstract

**Highlights:**

**What are the main findings?**

- Multi-horizon prediction provides useful early-warning signals only within limited near-term horizons.
- Threshold-based alarm policies exhibit a structural trade-off, where moderate detection requires disproportionately high alarm rates.

**What are the implications of the main findings?**

- Predictive accuracy alone is insufficient for deployment; decision-level behavior must be explicitly evaluated.
- Single-threshold alarm policies are inherently limited under severe class imbalance, requiring alternative decision strategies.

**Abstract:**

Photovoltaic (PV) inverter fault prediction is critical for maintaining system reliability and minimizing energy loss. While recent studies have improved predictive accuracy using data-driven approaches, most evaluations remain focused on offline settings and do not address how probabilistic predictions are translated into operational decisions. This study investigates multi-horizon fault prediction for PV inverters under real-world constraints, with a particular focus on decision-level behavior. A modular prediction framework is implemented by combining transformer-based TimeXer embeddings with probabilistic classification using XGBoost. The model operates on sliding-window sensor data and produces fault probabilities across multiple future horizons. To support operational use, these probabilities are aggregated into a single risk score, and threshold-based alarm policies are evaluated through a systematic threshold sweep. The results show that predictive performance varies across horizons, with usable lead-time information concentrated in near-term predictions. Under severe class imbalance, imbalance-aware training significantly improves detection performance in precision–recall space, but performance remains sensitive to temporal variation. Most importantly, the threshold-sweep analysis reveals a structural trade-off between detection performance and alarm burden, where achieving moderate early-warning capability requires substantially increased alarm rates. These findings indicate that improving predictive accuracy alone is insufficient for practical deployment. Instead, decision-level behavior must be explicitly considered when designing predictive maintenance systems under operational constraints.

## 1. Introduction

Photovoltaic (PV) power plants rely on inverters as critical components that convert direct current (DC) into alternating current (AC) and regulate interaction with the power grid. As a result, inverter faults can directly lead to energy yield loss, unplanned downtime, and safety risks. Ensuring reliable inverter operation is therefore essential for maintaining both system performance and economic viability in PV systems [1,2,3,4,5]. Recent review studies also show that PV fault analysis has expanded well beyond component-level diagnosis to include fault detection, predictive maintenance, intelligent maintenance, and reliability-oriented monitoring at the system and plant levels [6,7,8,9,10,11]. This broader literature suggests that practical PV maintenance problems should be understood not only in terms of identifying fault types, but also in terms of maintaining inverter reliability, supporting plant-level operation, and improving maintenance decision-making under real operating conditions.

Photovoltaic (PV) inverters are implemented in several configurations, including central, string, and module-level architectures, each involving different trade-offs in scalability, monitoring granularity, fault isolation, and maintenance strategy. In utility-scale and commercial PV plants, grid-connected inverters are especially important because they must not only convert direct current (DC) into alternating current (AC) but also maintain stable interaction with the power grid under changing operating and environmental conditions. As a result, inverter reliability depends not only on internal electrical conversion performance but also on thermal stress, control behavior, and grid-interaction dynamics.

From an operational perspective, PV inverter faults can be broadly grouped into electrical, thermal, control-related, and grid-interaction-related categories. Electrical faults may be reflected in abnormal DC input behavior, switching-stage instability, or AC-side imbalance. Thermal faults are associated with overheating and temperature-driven degradation, while control- and grid-related faults may appear through unstable output regulation, abnormal power factor behavior, or frequency-related response. Although the exact physical mechanisms differ across inverter designs and plant conditions, these fault categories commonly manifest through measurable changes in voltage, current, power, temperature, and grid-related telemetry.

This operational view is directly relevant to the present study because the dataset contains DC-side measurements, AC-side output variables, grid-related indicators, and operating-state variables. Together, these measurements provide complementary information on electrical state, thermal condition, grid interaction, and longer-term operational context. Accordingly, sensor-based fault prediction is not treated here as a purely offline pattern-recognition task, but as an operational forecasting problem in which multivariate telemetry must be translated into maintenance-relevant decisions under real-time constraints. This framing is consistent with recent PV literature that increasingly links fault detection to predictive maintenance workflows, grid-connected system reliability, and intelligent operation support rather than to isolated fault classification alone [7,8,10]. It also aligns with recent application-oriented studies on grid-connected fault detection, dynamic AI-based fault detection, and machine-learning-based plant monitoring under operational conditions [6,12,13]

Recent advances in data-driven methods have enabled fault prediction using sensor data collected from PV systems [14,15,16,17,18]. However, most prior studies have been conducted in offline settings or have focused on one-step forecasting [19,20,21], where the objective is to predict faults at a single future time point. These approaches differ significantly from real operational environments, where decisions must be made continuously based on streaming data [21], and where maintenance planning requires advance information across multiple future time horizons rather than a single-step estimate [19,21].

In practice, fault prediction systems are expected to provide continuously updated probabilistic estimates of future faults while operating under real-time constraints [15,19,22,23]. Moreover, these probabilistic outputs must be translated into actionable alarm decisions that support maintenance planning. This introduces an additional layer of complexity beyond prediction accuracy. In particular, the conversion of probabilistic forecasts into binary alarm signals involves a trade-off between early detection capability and false-alarm burden [24,25]. Excessive sensitivity may lead to frequent false alarms and operational inefficiency, whereas conservative thresholds may delay detection and reduce maintenance lead time. Despite its practical importance, this decision-level trade-off has not been systematically examined in the context of PV inverter fault prediction.

Real-world PV inverter data further complicate this problem due to several inherent challenges, including severe class imbalance, temporal uncertainty in fault onset, and non-stationarity arising from varying environmental and operating conditions [26,27]. These factors make it difficult to maintain stable predictive performance and reliable decision-making under deployment conditions. As a result, evaluating predictive models solely in terms of accuracy may not adequately reflect their practical usefulness in operational settings [22,26,28].

To address these challenges, this study investigates multi-horizon fault prediction for PV inverters from an operational perspective. A modular prediction pipeline is implemented by combining transformer-based TimeXer embeddings with XGBoost probabilistic classification. The model operates on sliding-window sensor data and produces fault probabilities across multiple future horizons, enabling real-time inference and forward-looking maintenance support.

Rather than focusing only on predictive performance, this study emphasizes how prediction outputs behave when translated into operational decisions. Specifically, threshold-based alarm policies are analyzed using a systematic sweep of threshold values, allowing the relationship between detection performance and alarm burden to be quantified under realistic constraints [24,25]. Through this analysis, the study reveals a fundamental trade-off structure that limits the effectiveness of conventional threshold-based approaches in practice.

The main contributions of this study are as follows. First, a real-time multi-horizon prediction framework is implemented and evaluated using operational PV inverter data under deployment-like conditions. Second, the study provides a systematic analysis of the trade-off between detection performance and alarm burden, highlighting practical limitations of single-threshold alarm policy designs under severe class imbalance. Third, complementary SHAP-based interpretability analyses are conducted for both a raw-window baseline and the proposed decision layer, allowing sensor-space attribution and decision-layer attribution to be examined separately. These findings provide practical insights into the design and deployment of predictive maintenance systems in sensor-driven energy infrastructures.

This study challenges that assumption by explicitly analyzing how predictive outputs behave when translated into alarm decisions under real-world constraints, while also separating input-level interpretability from decision-level interpretability through complementary baseline and decision-layer analyses.

## 2. Related Works

The Introduction highlighted the need for fault prediction systems that operate under real-world constraints, including real-time inference, multi-horizon probabilistic prediction, and decision-making through alarm policies. Prior work is therefore reviewed from four perspectives: (1) fault categories and signal- and feature-based approaches [3], (2) model-based methods [29], (3) deep-learning-based time-series learning [19,21], and (4) operational decision-making [24,25], including alarm policies and imbalance handling. Across these streams, a common limitation emerges: while predictive performance has improved, the translation of prediction outputs into reliable operational decisions remains insufficiently addressed [24,25]. Recent review papers reinforce this point from complementary perspectives. Some surveys emphasize PV fault detection techniques across electrical, thermal, signal-based, and AI-based approaches [11], whereas others focus more directly on predictive maintenance, intelligent maintenance, and reliability assessment in grid-connected PV systems [6,7,8,9,10]. Together, these studies show that PV monitoring research is increasingly moving toward operationally grounded formulations in which maintenance relevance, reliability, and deployability must be considered together.

From an operational perspective, PV inverter abnormalities can be broadly grouped into electrical, thermal, control-related, and grid-interaction-related categories. Early studies on PV inverter faults primarily focused on fault detection and diagnosis, identifying abnormal operating states using features derived from voltage, current, harmonic components, and ripple characteristics [3,15,16,30]. From an operational perspective, these studies can be understood as addressing mainly electrical abnormalities, while also capturing some thermal- or grid-related signatures through sensor-observable changes in inverter behavior [30]. These approaches, often combined with relatively simple classifiers such as k-nearest neighbors, demonstrated strong detection capability under controlled conditions [3,17]. However, their applicability in real-world environments is constrained by sensitivity to operating conditions and computational overhead associated with signal transformations [15,18]. More importantly, these methods are typically designed for state identification rather than forward-looking prediction, and do not directly support multi-step forecasting or integration with operational decision processes [19,21]. More recent application studies have also extended PV fault detection toward operational settings, including automated fault detection in grid-connected solar systems, dynamic AI-based fault detection using operational and environmental information, and machine-learning-based analysis of PV plant performance patterns [6,12,13]. These studies strengthen the practical relevance of data-driven monitoring, but they also highlight that field deployment requires greater attention to reliability, maintenance context, and operational decision support.

Data-driven subspace and statistical prediction methods have been explored to improve forecasting efficiency and reduce uncertainty under certain assumptions [20,31]. These approaches are useful when system behavior can be represented in a relatively structured form, but their effectiveness depends on how well those assumptions are maintained in operational settings. While these approaches can enhance predictive performance in structured settings, they are less effective in handling key characteristics of operational PV data, including non-stationarity, uncertainty in fault timing, and severe class imbalance [26,27,32]. In addition, many studies focus on point prediction at a single time horizon [19,21], which limits their usefulness for maintenance planning that requires advanced information across multiple future time steps.

Model-based approaches, grounded in physical system dynamics and control logic, have also been widely investigated [29,33]. Such methods remain important because they provide a physically interpretable view of inverter behavior and can be naturally linked to converter operation and control. These methods offer advantages in interpretability and computational efficiency, particularly when integrated with inverter control systems. However, their performance depends heavily on the validity of modeling assumptions and parameter estimation, and they often require additional effort to maintain robustness under changing operating conditions [26,27]. As a result, ensuring consistent performance under non-stationary environments and sparse fault labels remains a challenge.

Recent advances in deep learning, particularly recurrent and Transformer-based architectures, have enabled the modeling of complex temporal dependencies and long-range interactions in time-series data [19,21,34,35,36,37]. These methods have shown strong performance in various prediction tasks, including fault forecasting [14,19,28,35]. However, most studies evaluate performance in offline settings and focus on improving representation quality or prediction accuracy [19,21,35]. In operational environments, where data distributions evolve over time and fault events are rare, high predictive accuracy alone does not guarantee reliable system behavior [26,27]. In particular, the connection between probabilistic outputs and actionable decisions is often not explicitly addressed [24,25].

From a deployment perspective, predictive maintenance systems must convert probabilistic outputs into alarm signals that trigger maintenance actions [24,25]. A recent survey in IEEE Systems Journal further places maintenance and forecasting in power electronic systems within a broader AI-enabled system-of-systems perspective, highlighting fault/anomaly detection, remaining useful life estimation, and operational forecasting as interconnected lifecycle tasks rather than isolated algorithmic problems [38]. This introduces a critical trade-off between detection capability and false-alarm burden. While prior studies have recognized the importance of false-alarm control [24,25], many evaluations remain centered on model-centric metrics such as accuracy or AUC [28], without systematically analyzing how threshold-based alarm policies behave under realistic operational constraints. In addition, the practical value of a complex temporal model is difficult to assess when comparisons against simpler non-temporal baselines are not clearly presented. Consequently, systems with strong predictive performance may still generate excessive alarms or fail to provide timely warnings when deployed in practice.

Overall, existing research has advanced fault prediction across multiple methodological streams [3,19,21,29]. However, an important gap remains in understanding how predictive models perform when integrated into operational decision processes [24,25]. In particular, the interaction between multi-horizon prediction outputs, class imbalance, and threshold-based alarm policies under real-time constraints has not been systematically analyzed using operational data. This gap is especially important in PV inverter monitoring because practical maintenance systems must evaluate not only predictive discrimination but also the usability of the resulting alarm policy under operational constraints. To address this gap, this study investigates multi-horizon fault prediction in PV inverters from an operational perspective. Rather than focusing solely on predictive accuracy, the study analyzes how probabilistic forecasts are translated into alarm decisions and how this process affects practical usability. By combining representation learning, probabilistic classification, and threshold-based decision analysis within a unified experimental framework, the study provides insight into the structural limitations of conventional predictive maintenance pipelines under real-world conditions. In this respect, the present study is positioned not simply as a generic time-series prediction task, but as an operational PV inverter forecasting problem in which inverter-specific telemetry, rare-event fault structure, reliability considerations, and alarm-policy translation must be considered together. This positioning is consistent with recent review literature calling for stronger connections between fault analytics, maintenance planning, and system-level operational reliability in PV applications [7,8,10].

## 3. Methodology and Materials

### 3.1. Methodology Overview

**Figure 1:**
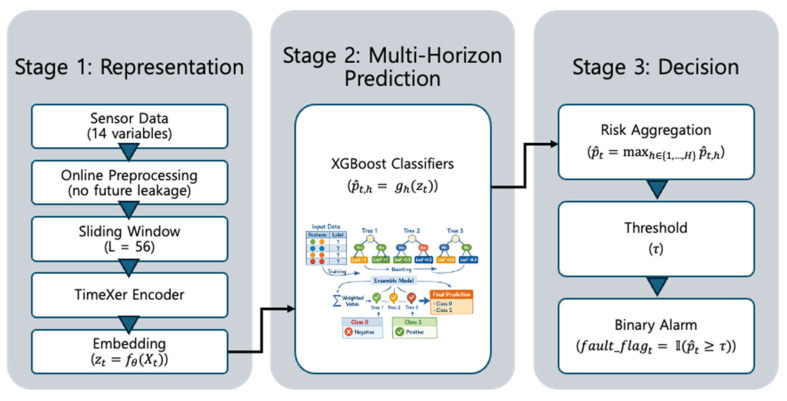
Overall framework of the proposed operation-aware multi-horizon fault prediction system for PV inverters.

This study examines multi-horizon fault prediction for photovoltaic (PV) inverters under operational constraints, focusing on how prediction outputs are translated into actionable alarm decisions. Figure 1 illustrates the overall framework of the proposed pipeline. The methodology is structured to reflect real deployment conditions, including streaming data processing, temporal consistency, and decision-making based on probabilistic outputs. The framework is organized into three stages: (i) representation learning, (ii) multi-horizon probabilistic prediction, and (iii) decision-making.

To support real-time operation, input data are constructed as sliding windows of fixed length (56 time steps) directly from streaming measurements. Preprocessing is restricted to online-compatible operations that do not rely on future information, including time alignment and forward filling with limited interpolation. Model parameters are fixed after training to ensure consistency during inference. These steps define the input preparation process for the proposed framework.

Fault prediction is formulated as a multi-horizon probabilistic task, where the model estimates fault likelihoods across 15 future time steps. This formulation enables the analysis of lead-time characteristics and allows prediction behavior to be examined across different horizons rather than at a single point. A Transformer-based time-series encoder is used to extract representations of temporal dependencies and cross-variable interactions from multivariate sensor data, which are then used as inputs to a probabilistic classifier. Together, these components constitute the representation learning and multi-horizon prediction stages of the framework.

To address class imbalance and temporal drift, the evaluation is conducted using time-ordered data splitting to prevent temporal leakage. Class-weighted learning is applied to improve minority-class detection. The model structure separates representation learning, classification, and decision layers, enabling the behavior of each component to be examined independently under changing data conditions.

Operational decisions are derived by converting multi-horizon probabilistic outputs into a single risk score, defined as the maximum probability across horizons. This aggregation reflects a conservative decision strategy in which a high risk at any future horizon can trigger attention. A threshold-based alarm policy is then applied to generate binary alarm signals. This defines the decision layer of the proposed framework, where multi-horizon prediction outputs are translated into a single operational alarm signal.

Rather than assuming a fixed threshold, the methodology evaluates a range of threshold values to analyze how detection performance and alarm burden vary under different operating conditions. This allows the trade-off between early detection and false alarms to be systematically characterized and provides insight into the limitations of threshold-based decision policies in practical deployment.

### 3.2. Materials: Operational Dataset and Feature Set

**Figure 2:**
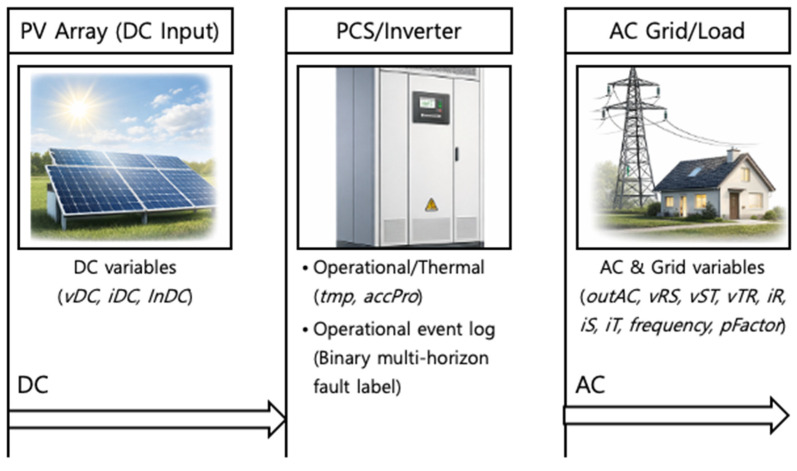
Conceptual configuration of the grid-connected PV inverter system and the associated measurements used in this study. The arrows indicate the main power-flow direction from the PV array DC input through the PCS/inverter to the AC grid/load.

This study uses operational sensor and telemetry data collected from photovoltaic (PV) inverters deployed in real plants. The dataset contains missing values, measurement noise, and temporal distribution shifts caused by changing environmental and operating conditions. Fault labels are highly imbalanced, reflecting the rarity of fault events in practice. Figure 2 presents a conceptual configuration of the monitored grid-connected PV inverter system and the available measurements used in this study. The variables are organized according to the DC-side input, inverter conversion and control stage, and AC-side/grid-interfacing output. This organization is intended to reflect the inverter as an operational power-electronic subsystem rather than to present the measurements as an undifferentiated sensor set. In a grid-connected PV inverter, the DC-side variables describe the incoming electrical condition supplied from the PV array, the conversion/control stage reflects the internal power-electronic operation and associated thermal or operational stress, and the AC-side/grid-interfacing variables represent output regulation, phase balance, and interaction with grid conditions. This interpretation is consistent with recent PV maintenance, reliability, and forecasting studies that emphasize subsystem-level operational understanding in grid-connected PV and power-electronic systems [7,8,10,38]. From this perspective, the inverter aspect of the present dataset lies not only in the presence of an inverter in the system diagram, but also in how the monitored variables correspond to distinct functional parts of inverter operation.

From a system perspective, the monitored inverter configuration can be interpreted as consisting of a DC-side input stage, an inverter conversion stage, and an AC-side/grid-interfacing output stage. Accordingly, the available telemetry variables capture complementary aspects of inverter operation across these functional parts of the system. More specifically, the DC-side variables (*vDC*, *iDC*, *InDC*) represent the electrical input condition delivered to the inverter, the thermal and operational variables (*tmp*, *accPro*) provide information on internal operating stress and accumulated usage context, and the AC-side/grid-related variables (*outAC*, *vRS*, *vST*, *vTR*, *iR*, *iS*, *iT*, *frequency*, *pFactor*) capture output-side regulation, phase behavior, and grid interaction. In inverter-oriented monitoring, abnormalities across these groups may indicate different but coupled precursor signatures, including unstable input behavior, thermal stress, conversion-stage irregularity, output-side imbalance, and grid-interaction-related response changes. Accordingly, the prediction task in this study is interpreted as forecasting maintenance-relevant precursor behavior across inverter subsystems rather than as modeling a generic multivariate sequence detached from inverter operation, which is also consistent with recent operational and predictive-maintenance-oriented PV literature [6,7,12,13].

Two data representations are used. First, an offline labeled window dataset is constructed for model development and evaluation. Second, a minute-level snapshot dataset is used to emulate streaming operations and to assess real-time inference behavior. Fault events are obtained from plant event logs and aligned with the corresponding sensor timestamps.

Sliding-window samples are constructed with a fixed window length of 56 time steps and 14 input variables. Multi-horizon binary labels are defined over 15 future time steps. The resulting dataset consists of 100,667 samples, each represented as a three-dimensional tensor with dimensions corresponding to sample count, window length, and feature variables. The output labels are organized as 15-dimensional binary vectors. The positive rate per horizon is approximately 0.17–0.19%, while the aggregated within-horizon label used for alarm evaluation has a positive rate of 2.6%.

Table 1 reports the prevalence of fault-positive windows in the labeled dataset. Only 1402 out of 749,304 labeled windows are positive, corresponding to 0.187% of the full dataset. This confirms that the task is highly imbalanced at the operational-window level. Table 2 summarizes the broad fault categories identified from the operational event log. Although multiple categories were observed, their prevalence relative to the full labeled dataset remains very small, and the category-specific distribution is highly uneven. Therefore, the present study does not formulate the task as multiclass fault diagnosis. Instead, the task is defined as binary multi-horizon fault forecasting, where the objective is to predict whether any fault event will occur within each future horizon. The counts in Table 2 are derived from event-log annotations and therefore summarize fault-label composition, whereas Table 1 reports prevalence at the labeled-window level after temporal alignment and window construction.

The input variables include electrical measurements on both the DC and AC sides of the inverter. DC-side variables consist of DC voltage (*vDC*), DC current (*iDC*), and DC input power (*inDC*). AC-side variables include output power (*outAC*), three-phase line-to-line voltages (*vRS*, *vST*, *vTR*), and phase currents (*iR*, *iS*, *iT*). Grid-related variables, such as frequency (*frequency*) and power factor (*pFactor*), are included to reflect grid interaction. In addition, accumulated energy production (*accPro*) and inverter temperature (*tmp*) are incorporated to represent long-term operational state and thermal conditions. Taken together, these variables provide observable information related to electrical behavior, thermal stress, and grid-interaction conditions, which are plausible carriers of pre-fault signatures in operational PV inverter systems.

The fault events recorded in the operational logs may arise from multiple underlying causes, including electrical abnormalities, thermal stress, control-related instability, or grid-interaction-related disturbances. However, the number of samples associated with individual fault categories was too small and too unevenly distributed to support reliable class-specific modeling. For this reason, the present study does not formulate the task as multiclass fault diagnosis. Instead, the task is defined as binary multi-horizon fault forecasting, where the objective is to predict whether any fault event will occur within each future horizon.

To emulate real-time deployment, a snapshot dataset is collected as daily CSV files with minute-level resolution. The dataset spans 23 consecutive days, from 26 December 2025 to 17 January 2026, and includes 24 inverter streams identified by plant and equipment identifiers across six plants. Each day contains up to 34,560 records, corresponding to 1440 time steps per inverter. Each record includes a timestamp and the same 14 variables used in the offline dataset. The timestamp is used as the primary temporal index for window construction to ensure consistent ordering. Minor schema differences across files are resolved using a deterministic column-mapping rule prior to analysis. The dataset spans 23 consecutive days, which limits the diversity of observed fault conditions and long-term variability.

All variables are used in their original multivariate time-series form without additional feature engineering. Missing or inconsistent values are handled through time-aligned preprocessing under online-compatible constraints.

### 3.3. Target Definition: Real-Time Multi-Step Fault Forecasting

In operational settings, fault prediction requires not only identifying current faults but also estimating the likelihood of future fault events. Accordingly, the task is formulated as a multi-step probabilistic prediction problem. For each time index t, the framework predicts fault occurrence over multiple future horizons rather than producing a single one-step forecast.

At time t, the input $X_{t}$ is defined as a multivariate time series over the most recent window of length L  with F variables. For each future step h∈{1,…,H}, a binary label $y_{t,h}$ indicates whether a fault occurs at that future time. The model estimates the conditional probability of fault occurrence for each horizon, producing a set of horizon-wise probabilities. These horizon-wise labels serve as the primary learning targets of the model and are used to evaluate lead-time-dependent predictive performance.

To support operational use, the horizon-wise probabilities are aggregated into a single risk score. The aggregation is defined as the maximum probability across all horizons, representing the highest estimated risk within the prediction window. This risk score is compared with a threshold to generate a binary alarm indicator at time t. For operational alarm evaluation, an additional aggregated reference label is defined to indicate whether any fault occurs within the full prediction horizon. This label is used only for decision-oriented evaluation after risk aggregation and should be distinguished from the horizon-wise labels used for model learning.

This distinction is important because the prevalence of the aggregated within-horizon label is naturally higher than that of the individual horizon-wise labels. In the present dataset, the positive rate of each horizon-wise label is approximately 0.17–0.19%, whereas the aggregated label used for alarm evaluation has a positive rate of 2.6%. This difference arises from the label definition rather than from any inconsistency in the data.

### 3.4. Online-Compatible Preprocessing and Window Construction

Operational sensor data streams contain missing values, irregular time stamps, and occasional abnormal readings. All preprocessing steps are designed to be compatible with real-time deployment. Preprocessing is therefore designed to ensure temporal consistency and to avoid the use of future information. Time alignment is applied to synchronize all variables to a common sampling interval. Missing values are handled using forward filling and time-based interpolation where necessary. When extreme values are present, optional outlier handling methods such as range limiting or scaling can be applied to reduce their influence. Sliding-window inputs are constructed such that, at each time t, the input consists of the most recent 56 time steps. For each window, horizon-wise binary labels are generated across all forecast horizons. For alarm evaluation, an additional operational target is defined. Horizon-wise probabilities are aggregated into a single risk score using the maximum across horizons, and a corresponding reference label is defined to indicate whether any fault occurs within the prediction window. To prevent temporal leakage, all preprocessing steps are restricted to information available up to time t. All transformations are therefore applied in a strictly forward-looking manner. No smoothing or filtering methods that require future observations are used. When normalization or scaling is applied, parameters are estimated using only the training period and then applied unchanged to validation and test periods.

### 3.5. Proposed Method: Modular Hybrid Pipeline for Multi-Horizon Fault Prediction

The proposed approach consists of three components: (i) representation learning from multivariate time-series inputs, (ii) probabilistic classification for multi-horizon fault prediction, and (iii) threshold-based alarm generation. These components are implemented sequentially and can be evaluated independently. This modular design explicitly separates representation learning, probabilistic prediction, and decision-making, allowing each component to be analyzed under operational constraints.

#### 3.5.1. Stage 1: Transformer-Based Time-Series Encoder

The first stage extracts a fixed-length embedding from a multivariate input window. Given an input sequence $X_{t}$, the encoder produces an embedding $z_{t}$ defined as:

(1) $$z_{t}=f_{\theta }(X_{t})$$

where $f_{\theta }$ denotes the parameterized encoder.

A Transformer-based architecture (TimeXer) is used to capture temporal dependencies and interactions across variables in the input sequence. The encoder is trained during the training phase and then fixed for embedding extraction during inference. The resulting embeddings are used as inputs to the classification stage. Rather than directly performing final fault classification, this stage is used to learn a compact latent representation that summarizes temporal precursor patterns in the multivariate window.

#### 3.5.2. Stage 2: Probabilistic Classification for Multi-Horizon Forecasting

The second stage estimates fault probabilities for multiple future horizons based on the embedding. For each horizon h∈{1,…,H}, a separate probabilistic classifier is trained:

(2) $${\hat{p}}_{t,h}=g_{h}(z_{t})$$

where $z_{t}$ is the embedding vector extracted from the input window at time *t*, $g_{h}(\cdot )$ is the probabilistic classifier corresponding to forecast horizon *h*, ${\hat{p}}_{t,h}$ is the estimated probability of a fault occurring at horizon *h*.

XGBoost is used as the classification model. Class imbalance is addressed through class weighting during training. A separate model is trained for each horizon to allow horizon-specific behavior to be captured. This horizon-wise design allows different lead times to have different decision boundaries, while maintaining a lightweight inference structure after representation extraction.

#### 3.5.3. Stage 3: Threshold-Based Alarm Generation

To support operational decision-making, horizon-wise probabilities are aggregated into a single risk score:

(3) $${\hat{p}}_{t}=\underset{h\in \{1,\ldots ,H\}}{max}{\hat{p}}_{t,h}$$

A threshold τ  is applied to generate a binary alarm signal:

(4) $${\mathrm{fault}\_\mathrm{flag}}_{t}=I({\hat{p}}_{t}\geq \tau )$$

where ${\hat{p}}_{t}$ is the aggregated risk score at time *t*, computed as the maximum probability across all horizons, and I(·) is the indicator function that returns 1 if the condition is satisfied and 0 otherwise. The resulting binary variable ${\mathrm{fault}\_\mathrm{flag}}_{t}$ indicates whether an alarm is triggered at time *t*. This stage defines the decision layer of the framework, in which multi-horizon probabilistic outputs are translated into a single operational alarm signal.

To examine decision behavior under different operating conditions, performance is evaluated across a range of threshold values. This enables the relationship between detection performance and alarm burden to be analyzed systematically. The threshold used for evaluation is selected on a validation set and then fixed for testing.

Two alarm-related metrics are considered. The alarm rate is defined as the proportion of time steps at which an alarm is issued. The false-alarm rate is defined as the proportion of alarms that are not associated with a fault event, based on a consistent counting scheme. Because the proposed framework separates probabilistic prediction from threshold-based decision-making, the operational behavior of the alarm policy can be examined independently of the underlying predictive model.

### 3.6. Training Strategy: Imbalance Handling and Real-Time Constraints

Operational inverter data exhibit a strong class imbalance, with positive (fault) instances accounting for approximately 2.6% of samples. To address this, training is performed using a combination of class-weighted learning and training-set-only oversampling under the aggregated operational label used for alarm-oriented evaluation.

Class imbalance is handled using cost-sensitive learning, where the contribution of positive samples is increased through the positive-class weighting option in XGBoost. This weighting is applied during training while preserving the original data distribution. In addition, minority-class augmentation is performed using the Synthetic Minority Over-sampling Technique (SMOTE), applied only to the training set. Oversampling is conducted in a transformed feature space rather than directly on raw time-series sequences, in order to avoid generating unrealistic synthetic trajectories in the original temporal domain. Each input window is first represented as either an embedding vector (e.g., TimeXer embedding) or a derived feature vector, and synthetic samples are generated in this representation space. No oversampling is applied to validation or test data.

Data splitting follows a chronological order to prevent temporal leakage. To further reduce information overlap between training and evaluation segments, a buffer interval is introduced at split boundaries. The buffer length is set to 55 time steps, corresponding to the window overlap, and ensures that adjacent windows do not share information across splits. All model parameters, including class weights and preprocessing settings, are determined using the training data and validation period. The selected configuration is then fixed for evaluation on the test period.

### 3.7. Evaluation Protocol: Operation-Aware Metrics

Evaluation is conducted using both threshold-independent probabilistic metrics and threshold-dependent operational metrics. To avoid ambiguity, performance is reported separately at three levels: horizon-level probabilistic prediction, window-level alarm classification, and event-level operational detection.

For multi-horizon evaluation, performance is computed separately for each forecast horizon. For each horizon h∈{1,…,H}, receiver operating characteristic area under the curve (ROC-AUC) and precision–recall area under the curve (PR-AUC, reported as average precision) are calculated. In addition, an aggregated operational risk score is evaluated by combining horizon-wise probabilities using the maximum across horizons. Probabilistic performance for this aggregated score is reported using ROC-AUC and PR-AUC. At a selected threshold, classification metrics including precision, recall, and F1-score are computed. At a selected threshold, precision, recall, and F1-score are computed at the window level using the aggregated operational label defined in Section 3.3. These metrics treat each time step as an independent evaluation unit.

Alarm-policy evaluation is performed by computing precision, recall, alarm rate, and false-alarm rate across a range of threshold values. Unless otherwise stated, threshold-sweep trade-off analyses in this study are reported at the window level rather than at the event level. These metrics are used to construct trade-off curves that characterize the relationship between detection performance and alarm burden. The threshold used for evaluation is selected on the validation set and then fixed for testing.

Data splitting follows a chronological order. Training, validation, and test periods are defined explicitly in the experimental setup. To reduce information overlap caused by sliding-window sampling, a buffer interval is introduced at split boundaries, consistent with the window length. Event-level metrics are reported separately when operational episode analysis is required. In this setting, each fault event is treated as a single instance, and metrics such as event recall and lead time to first alarm are computed independently from the window-level classification metrics. These event-level results are not directly comparable to window-level precision, recall, or F1-score and are therefore presented separately.

## 4. Experiments

This section presents the experimental setup used to analyze multi-horizon fault prediction under operational conditions. The experiments are designed to examine prediction behavior, robustness under imbalance, real-time feasibility, and decision characteristics when probabilistic outputs are converted into alarm signals. All experiments follow a chronological evaluation protocol to prevent temporal leakage. Model training is performed using past data, while validation and test sets represent future periods. Preprocessing parameters are estimated on the training data and applied unchanged during evaluation.

Given the severe class imbalance at the operational-window level, performance is assessed using both threshold-independent and threshold-dependent metrics. Under the aggregated operational label used for alarm-oriented evaluation, positive samples account for approximately 2.6% of the data. Probabilistic performance is evaluated using ROC-AUC and PR-AUC, while operational behavior is examined using metrics such as recall, precision, and alarm rate under varying threshold conditions. As described in Section 3.7, these metrics are interpreted at different evaluation levels, including horizon-level probabilistic prediction, window-level alarm classification, and event-level operational detection, depending on the purpose of each experiment.

The experiments are organized into four components (E1–E4), each focusing on a different aspect of the system. These include real-time inference behavior, multi-horizon prediction characteristics, robustness under class imbalance and temporal variation, and alarm-policy behavior under operational constraints. E1 evaluates whether the proposed framework is feasible for real-time deployment. E2 examines how predictive performance changes across future horizons. E3 evaluates robustness under imbalance and temporal variation, including comparison with simpler baselines and ablated variants. E4 analyzes how probabilistic outputs behave after aggregation and thresholding when translated into operational alarms. Multi-horizon prediction refers to estimating binary fault probabilities across multiple future time steps. The behavior of these horizon-wise predictions and their aggregated form is analyzed in relation to operational decision-making. Together, E1–E4 are designed to separate predictive performance from operational decision behavior, which is the central experimental objective of this study.

### 4.1. (E1) Real-Time Deployability Test

Experiment E1 evaluates whether the proposed pipeline can operate under real-time streaming conditions without introducing processing delays. The objective of this experiment is to characterize the computational behavior of the system in a deployment-like setting, rather than to assess predictive performance. Accordingly, E1 focuses on inference-time efficiency under streaming operation and does not evaluate discrimination quality or alarm accuracy.

To emulate real-time operation, inference is performed sequentially for each incoming time step, and the processing time is measured on a per-sample basis. The evaluation is conducted using the minute-level snapshot stream in chronological order so that each new sample is processed as if it were arriving online. The total computation is decomposed into four stages: (i) online preprocessing, including time alignment, missing-value handling, and window construction; (ii) embedding extraction using the TimeXer encoder; (iii) probabilistic inference using the XGBoost classifier to produce horizon-wise probabilities; and (iv) post-processing for operational decision-making, including aggregation of horizon-wise probabilities and threshold-based alarm generation.

Deployment performance is quantified using latency, throughput, and model footprint. Latency is defined as the processing time per sample and is summarized using mean, variability, and high-percentile statistics (e.g., 95th and 99th percentiles). Throughput is measured as the number of samples processed per second. Model footprint is reported using model file size and, where available, memory usage during inference. In addition to per-sample latency, E1 considers whether the measured runtime is sufficiently small relative to the one-minute sampling interval of the incoming stream. In addition, stage-wise runtime is analyzed to quantify the contribution of each component to the overall computational cost.

Because the monitored system consists of multiple inverter streams, the runtime results are interpreted from an operational perspective in terms of whether continuous sequential processing can be sustained without backlog under deployment-like conditions. This evaluation provides a basis for assessing whether the pipeline can sustain continuous operation under streaming conditions and identifies the dominant sources of computational overhead.

### 4.2. (E2) Multi-Horizon Lead-Time Prediction Test

Experiment E2 examines how predictive performance evolves across multiple forecast horizons and how this behavior affects the availability of maintenance lead time. At each forecast horizon, the model produces a fault probability corresponding to a future time step. These probabilities are evaluated independently against binary fault labels defined at each horizon. The task remains binary classification at each horizon, with multiple predictions generated simultaneously for different lead times. Accordingly, E2 focuses on horizon-level probabilistic prediction rather than on aggregated alarm behavior.

Performance is assessed using receiver operating characteristic area under the curve (ROC-AUC) and precision–recall area under the curve (PR-AUC, reported as average precision) for each horizon. The results are organized along the horizontal axis to characterize how predictive performance changes as the lead time increases. This evaluation allows the relationship between prediction horizon and performance to be examined explicitly. Because each horizon is evaluated against its own binary reference label, E2 isolates changes in horizon-wise predictive discrimination before any risk aggregation or threshold-based alarm conversion is applied. In particular, it enables identification of horizon ranges where predictive performance is maintained and where it degrades, providing insight into the effective lead-time window in which predictions remain operationally useful. In this context, “operationally useful” refers to horizons at which probabilistic discrimination remains sufficiently informative to support downstream alarm generation, rather than implying that a fixed alarm policy is already optimal at those horizons.

### 4.3. (E3) Robustness Under Class Imbalance and Temporal Variation

Experiment E3 evaluates whether fault detection performance remains reliable under two key operational challenges: severe class imbalance and temporal variation in data distribution. In addition, E3 examines whether the proposed framework remains robust when compared with simpler baselines and reduced variants of the hybrid architecture.

The dataset contains approximately 2.6% positive samples, representing rare fault events. Here, the 2.6% prevalence refers to the aggregated operational label used for alarm-oriented evaluation. To examine the effect of imbalance handling, four training configurations are compared using the same evaluation period. The baseline configuration applies standard training without explicit imbalance adjustment. The cost-sensitive configuration applies class-weighted learning. The oversampling configuration applies SMOTE in the representation space using only training data. The combined configuration applies both class weighting and training-only oversampling. All configurations are evaluated using an identical test split to ensure comparability. To further assess the contribution of the hybrid design, E3 also includes comparison with simpler baseline and ablated variants, such as non-hybrid or reduced aggregation settings, under the same evaluation protocol.

To assess temporal variation, the test period is divided into time blocks (e.g., weekly or predefined intervals), and performance is evaluated independently for each block. This enables analysis of whether model performance remains consistent over time or varies across different operating conditions. Performance is evaluated using metrics appropriate for imbalanced data. PR-AUC (reported as average precision) is used as the primary probabilistic metric. In addition, recall is evaluated under constraints on the alarm rate to reflect operational conditions. Variability across time blocks is summarized using measures such as the range or dispersion of performance metrics. This block-wise analysis is intended to reveal whether performance degrades or remains stable under temporally varying deployment conditions rather than to estimate a single pooled score alone.

This experiment examines both the effect of imbalance handling strategies and the stability of model performance under temporal variation. By combining imbalance-handling comparison, baseline/ablation analysis, and block-wise temporal evaluation, E3 provides a robustness-oriented assessment of the proposed framework under operationally realistic conditions.

As an additional simple temporal baseline, an LSTM classifier was evaluated under the same rare-event setting. The baseline used the same sequential NPZ input structure, with a many-to-one architecture consisting of a single LSTM layer (hidden dimension = 64) followed by a linear output layer producing a single binary logit. The target was defined either as the aggregated any-horizon label, using the maximum across horizons, or as a selected single horizon depending on the experiment. Data were split chronologically into training, validation, and test segments with ratios of 0.7, 0.1, and 0.2, respectively. Training used Adam (learning rate = 1 × 10^−3^), batch size 256, and up to 30 epochs with early stopping based on validation PR-AUC (patience = 5). Class imbalance was handled through positive-class weighting in the binary cross-entropy loss, and no additional oversampling was applied.

### 4.4. (E4) Threshold-Sweep Alarm Policy Test

Experiment E4 examines how probabilistic predictions are translated into operational alarm decisions and how this process behaves under different threshold settings. In operational systems, model outputs must be converted into a single alarm signal. Horizon-wise probabilities are therefore aggregated into an operational risk score, and a threshold is applied to generate a binary alarm at each time step. For evaluation, a within-horizon reference label is used to indicate whether a fault occurs within the prediction window. Accordingly, E4 evaluates the behavior of the alarm policy after risk aggregation and thresholding, rather than the horizon-level probabilistic predictions themselves.

To analyze decision behavior, performance is evaluated across a range of threshold values using a validation-to-test procedure. During the validation period, metrics are computed over a grid of thresholds, and feasible operating regions are identified under constraints such as maximum allowable alarm rate or minimum precision. A single threshold is then selected and fixed for evaluation on the test period. Operational performance is evaluated using precision, recall, F1-score, alarm rate, and false-alarm rate. Unless otherwise stated, these threshold-dependent metrics are computed at the window level using the aggregated operational reference label defined in Section 3.3. Results are presented as trade-off curves showing how detection performance and alarm burden vary with the threshold. This analysis characterizes how threshold-based decision policies behave under operational constraints. In particular, it reveals the extent to which improvements in detection performance require increased alarm rates and highlights limitations in achieving both high detection sensitivity and low alert burden simultaneously. Event-level quantities, such as episode recall or lead time to first alarm, are treated separately from the window-level threshold-sweep metrics and are reported only when explicitly stated.

## 5. Results

### 5.1. (E1) Real-Time Inference Latency

Deployment feasibility in a streaming environment was assessed by benchmarking end-to-end inference latency for the proposed TimeXer–XGBoost pipeline using a minute-level snapshot stream. Inference was conducted on a CPU-based desktop environment running Microsoft Windows 11 Home (Version 10.0.26100, Build 26100) with a 12th Gen Intel(R) Core(TM) i9-12900K processor (16 cores, 24 logical processors, 3.187 GHz) and 128 GB RAM (Intel, Santa Clara, CA, USA). Additional hardware and software details are summarized in Table 3. For each inverter stream across 24 inverter streams (unique plant–equipment pairs), sliding windows were constructed from the most recent 56 time steps, and probabilistic forecasts were produced for 15 future steps. Latency was measured per window and decomposed into four stages: online preprocessing, TimeXer embedding extraction, XGBoost inference, and post-processing. To mitigate initialization effects, an initial warm-up segment of 200 windows was excluded, and the remaining windows were summarized for each stream up to 5000 windows per stream.

On CPU across the 24 streams, the end-to-end latency showed a median of the per-stream mean latency of 1.10 ms, with the per-stream range spanning from 1.01 ms to 1.59 ms. Tail latencies remained low, with a median 95th-percentile latency of 1.30 ms (range: 1.16 ms to 1.64 ms) and a median 99th-percentile latency of 1.47 ms (range: 1.28 ms to 2.89 ms). Throughput ranged from 447 to 628 windows per second (median: 585 windows per second), indicating that per-window inference remained far below the one-minute sampling interval of the incoming stream.

**Figure 3:**
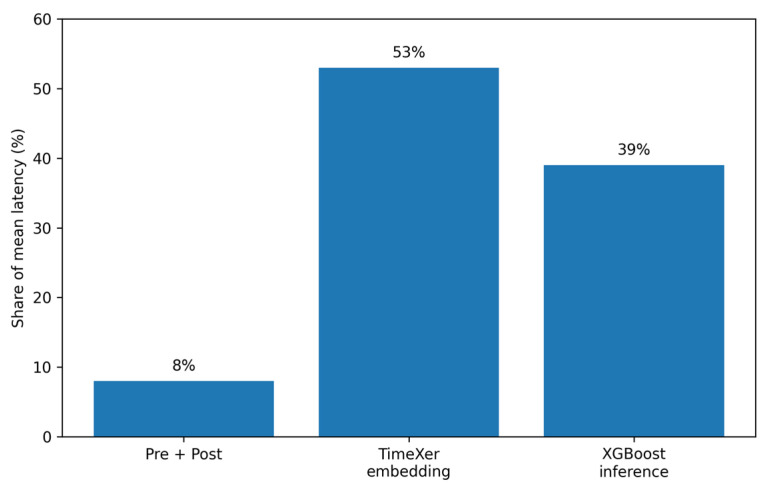
Stage-wise contribution to mean end-to-end latency.

Stage-wise decomposition shows that embedding extraction and classifier inference account for most of the runtime, contributing approximately 53% and 39% of the mean latency, respectively, while preprocessing and post-processing together account for only about 8% (Figure 3, Table 4). Thus, the dominant computational cost arises from representation extraction and probabilistic prediction rather than from online data handling or alarm conversion. These results indicate that inference latency remains within a millisecond scale under the evaluated conditions. Detailed latency statistics, including stage-wise runtime, are summarized in Table 4.

### 5.2. (E2) Multi-Horizon Lead-Time Prediction

Experiment E2 evaluates how predictive performance evolves across forecast horizons and how this affects the availability of maintenance lead time. Although predictions are generated for multiple future time points, targets remain binary at each horizon, indicating whether a fault event occurs at that specific lead time. For each horizon h, probabilistic performance is computed by comparing the predicted fault probability ${\hat{p}}_{t,h}$ with the corresponding binary label $y_{t,h}$. Performance is summarized using ROC-AUC and PR-AUC (average precision), and results are arranged along the horizon axis to characterize performance changes with increasing lead time. Consistent with the definition of E2, this analysis is performed at the horizon level before any risk aggregation or threshold-based alarm conversion is applied.

Table 5 reports the horizon-wise results at representative steps {1, 3, 5, 10, 15}, while the full horizon-wise trend is analyzed across all forecast steps. The positive rate remains extremely low and stable across horizons (approximately 0.0017–0.0019), confirming severe class imbalance throughout the lead-time setting. Despite this, the model maintains reasonably stable ranking performance, with ROC-AUC ranging from 0.756 to 0.829, peaking at h=5 and remaining above 0.75 even at the longest horizon (h=15). In contrast, PR-AUC values are lower in magnitude due to the rarity of positive samples but exhibit clear sensitivity to the prediction horizon. The highest PR-AUC is observed at h=1(0.0828), followed by a noticeable decrease at longer horizons (e.g., 0.0276 at h=3, 0.0368 at h=5), and remaining in a lower range 0.025–0.028 for h≥10.

These results indicate that short horizons provide stronger precision–recall performance, while longer horizons retain moderate discriminative ability in terms of ranking. This suggests that the near-term horizons contain the most informative fault signals for downstream decision-making, whereas longer horizons remain useful mainly in the sense of probabilistic ranking rather than strong positive-class retrieval. Accordingly, E2 does not claim that a fixed alarm policy is already optimal at short horizons; rather, it shows that the informativeness of horizon-wise probabilities decreases as the prediction horizon becomes longer.

**Figure 4:**
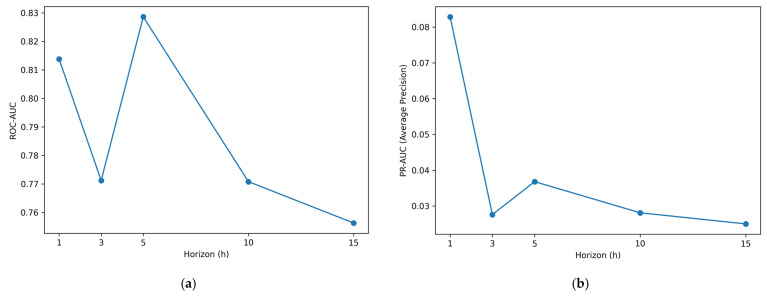
Horizon-wise probabilistic performance across forecast steps: (**a**) ROC-AUC and (**b**) PR-AUC (average precision). ROC-AUC remains relatively stable across horizons, while PR-AUC decreases with increasing lead time due to severe class imbalance.

To provide a complete view of performance variation across all forecast horizons, horizon-wise ROC-AUC and PR-AUC values are further illustrated in Figure 4. These figures present the evolution of probabilistic discrimination as a function of lead time.

As shown in Figure 4a, ROC-AUC values remain relatively stable across horizons, with moderate variation but no abrupt degradation as the prediction horizon increases. This indicates that the model preserves ranking ability even at longer lead times. In contrast, Figure 4b shows that PR-AUC decreases more noticeably as the horizon increases, reflecting the increasing difficulty of correctly identifying rare fault events at longer lead times under severe class imbalance.

These results highlight the distinction between ranking performance and positive-class retrieval. While the model maintains discriminative capability in terms of ranking across horizons, the precision–recall trade-off becomes more challenging as the prediction horizon increases. Importantly, these results correspond to horizon-wise probabilistic predictions before any risk aggregation or threshold-based alarm conversion, and therefore should be interpreted separately from the alarm-policy evaluation in E4.

### 5.3. (E3) Robustness Under Class Imbalance and Temporal Shift

Experiment E3 evaluates whether minority-class detection remains reliable under two operational challenges: (i) severe class imbalance and (ii) temporal distribution shift. The results are reported in two parts: (a) the effect of imbalance-handling strategies under a fixed evaluation setting, and (b) performance variability across time under operational conditions. In addition, E3 includes comparison with simpler baselines and reduced variants of the proposed hybrid framework in order to assess whether the observed gains remain robust under different model configurations.

Using the offline labeled window dataset (100,667 windows), imbalance-handling strategies are compared under a positive rate of approximately 2.6%. Here, the 2.6% prevalence refers to the aggregated operational label used for alarm-oriented evaluation. Because ROC-AUC can remain high under severe imbalance, PR-AUC (average precision) is used as the primary metric. Four training configurations are evaluated on the same test set: a baseline XGBoost model without imbalance handling, and three variants using class weighting, training-only oversampling in a representation space, and their combination (Table 6).

The results also show substantial improvements in precision–recall performance when imbalance-aware strategies are applied. The baseline model achieves a PR-AUC of 0.366, while class weighting and training-only oversampling increase PR-AUC to 0.554 and 0.558, respectively. The combined configuration yields a slightly lower PR-AUC of 0.542, indicating that applying multiple imbalance-handling strategies simultaneously does not necessarily lead to additional gains. In contrast, ROC-AUC remains around 0.90 across all configurations, suggesting that improvements are concentrated in the precision–recall regime. This pattern indicates that robustness gains under severe imbalance are expressed more clearly in minority-class retrieval than in ranking-based discrimination alone.

To further examine whether the observed robustness persists under matched evaluation coverage, a common-tail comparison was conducted between the proposed hybrid framework and a simpler raw XGBoost baseline. In this comparison, both models were evaluated on the same temporally aligned subset so that coverage differences would not confound the comparison. The results are summarized in Table 7. In Table 7, the same numerical threshold (τ = 0.5) was applied to both the raw baseline score and the proposed decision score. However, this should not be interpreted as a calibration-matched operating-point comparison, because the two scores are produced by different scoring pipelines and therefore do not necessarily share the same probability scale or threshold meaning.

**Figure 5:**
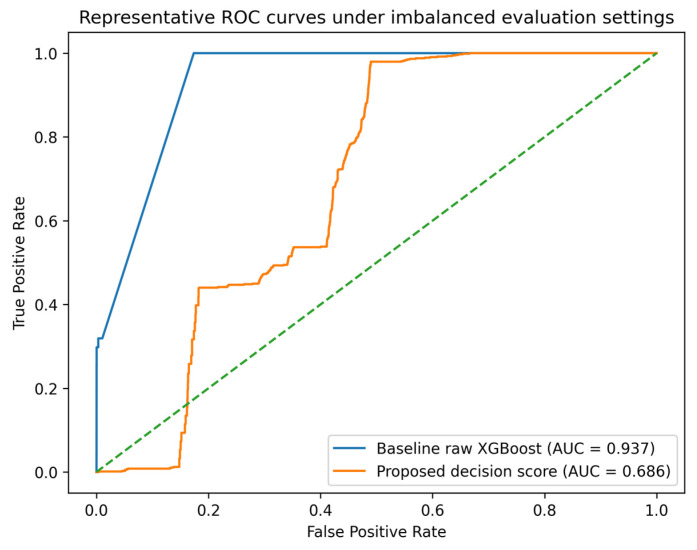
Representative ROC curves for the raw XGBoost baseline and the proposed decision score under highly imbalanced conditions.

To complement the confidence interval summaries in Table 8, a representative ROC-curve comparison is shown in Figure 5. The figure is intended to provide a visual reference for classifier behavior under highly imbalanced conditions rather than a strictly matched head-to-head comparison, because the baseline and proposed curves are derived from different evaluated subsets.

As shown in Figure 5, the raw XGBoost baseline exhibits stronger ROC discrimination on its evaluated subset, whereas the proposed decision score shows lower ROC-AUC on a different operational subset. Accordingly, this comparison should be interpreted as an uncertainty-aware visual reference rather than as direct evidence of superiority. Its main purpose is to supplement the PR-oriented analyses emphasized elsewhere in the manuscript with a reviewer-requested ROC-based visualization.

Under this fixed-cutoff common-tail setting, the proposed framework produced substantially more alarms than the baseline at τ = 0.5, while the resulting F1-score remained lower. This pattern suggests that the proposed decision score is more threshold-sensitive at this cutoff, which is consistent with a score-distribution or calibration difference relative to the raw baseline score. Accordingly, Table 7 is intended to illustrate that transferring the same numerical threshold across heterogeneous scores can yield misleading operational comparisons, rather than to claim superiority of one model over the other at a shared cutoff. For this reason, calibration-aware operating-point analysis is reported separately in the threshold-sweep evaluation in Section 5.4.

As an additional simple temporal baseline, an LSTM classifier was evaluated under the same rare-event setting using the positive-containing interval of the 23-day operational dataset. The model used a many-to-one architecture with a single LSTM layer (hidden dimension = 64) followed by a linear output layer, and was trained under a chronological train/validation/test split (0.7/0.1/0.2). Optimization used Adam with a learning rate of 1 × 10^−3^, batch size 256, and up to 30 epochs with early stopping based on validation PR-AUC (patience = 5). Class imbalance was handled through positive-class weighting in the binary cross-entropy loss, without additional oversampling. Under this setting, the model converged to near-random behavior, with ROC-AUC close to 0.5 and PR-AUC approximately equal to the positive prevalence (Table 9). This result indicates that a simple recurrent temporal baseline may fail to learn useful fault-discriminative structure under the evaluated rare-event regime.

A further comparison was conducted between a single-horizon classifier for the farthest forecast step (h=15) and the aggregated any-horizon target on the same positive-containing interval. The single-horizon model achieved a slightly higher ROC-AUC (0.975) than the any-horizon setting (0.963), but its PR-AUC was lower (0.334 vs. 0.424). This difference should be interpreted in light of the much lower positive prevalence at the single horizon (0.00010 vs. 0.00107). The result suggests that although a distant single-horizon target can remain rank-separable, the aggregated multi-horizon target is more informative in precision–recall terms under extreme event sparsity and is therefore more relevant for operational decision support. The corresponding quantitative comparison is summarized in Table 10.

An ablation analysis was also performed to examine whether the robustness characteristics can be attributed to specific components of the hybrid architecture. The full model was compared with reduced variants, including XGB-only, TX-max-only, and TX-mean-only settings. Under the current E3 summary setting, these variants exhibit nearly identical aggregate results. This outcome suggests that the contribution of individual components is not clearly separable at this summary level. Instead, the robustness behavior should be interpreted together with the baseline comparison and temporal variation analysis rather than as an isolated architectural effect.

**Figure 6:**
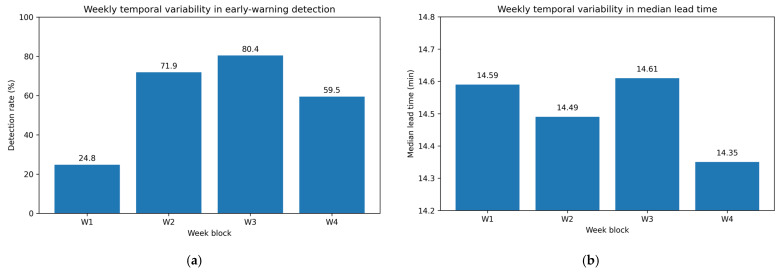
Weekly temporal variability in early-warning performance over the 23-day evaluation period: (**a**) detection rate and (**b**) median lead time.

To assess temporal variation, the evaluation is extended to a 23-day snapshot dataset, where early-warning detection performance is computed in a block-wise manner. Using a fixed operating threshold (τ=0.5) and a lead window of 15 min, detection performance is tracked across daily and weekly intervals. Weekly detection rates vary across blocks (e.g., 24.8% in W1, 71.9% in W2, 80.4% in W3, and 59.5% in W4), indicating substantial temporal variability under changing operating conditions (Table 11). This variability is also visualized in Figure 6a, which makes the instability across operational blocks more explicit. This block-wise analysis shows that operational performance cannot be fully characterized by a single pooled score and must be interpreted in relation to time-varying deployment conditions. Moreover, the median lead times remain close to the 15 min prediction window across weekly blocks, indicating that alarms tend to occur near the end of the available lead window rather than substantially earlier, as shown in Figure 6b. This limits the practical flexibility of intervention even when an event is successfully detected.

These results show that while imbalance-aware training improves minority-class detection, performance is not stable over time, reflecting the influence of temporal distribution shift in operational environments. Taken together with the simpler temporal baseline and the single-horizon comparison, the E3 results indicate that robustness must be assessed jointly with respect to imbalance handling, target definition, model structure, threshold sensitivity, and temporal variation rather than from a single aggregate metric alone.

### 5.4. (E4) Threshold-Sweep Alarm Policy Results

Two complementary operational views are reported in this section: (i) an episode-level detection–alarm trade-off over the snapshot replay using representative threshold values for interpretability, and (ii) a validation-selected operating point reported with window-level precision, recall, and F1-score under an alarm-rate budget (Table 12). These two views are reported separately because they correspond to different evaluation levels and should not be interpreted as directly interchangeable.

For the episode-level threshold-sweep analysis, fault episodes were first identified from the operational event log over the 23-day replay period. A total of 1196 episodes were recorded. Among these, 1041 episodes had prediction streams available for the full alarm-evaluation procedure and were retained for the threshold-sweep analysis, whereas episodes without matched prediction streams were excluded from episode-level evaluation. An episode was counted as detected if at least one alarm occurred within the 15 min lead window before the recorded event onset. Alarms occurring after the event onset were not counted as early-warning detections. When lead windows overlapped across adjacent events, each logged event was retained as a separate operational episode, and detection was evaluated with respect to its own pre-onset lead window.

Experiment E4 converts probabilistic risk outputs into an operational alarm policy and evaluates how detection performance varies with the decision threshold τ. Alarm burden is quantified using the alarm rate, defined as the fraction of time points flagged as alarms. Unless otherwise stated, the threshold-sweep results described below refer to the episode-level detection–alarm trade-off, whereas Table 12 reports validation-selected operating points using window-level classification metrics.

**Figure 7:**
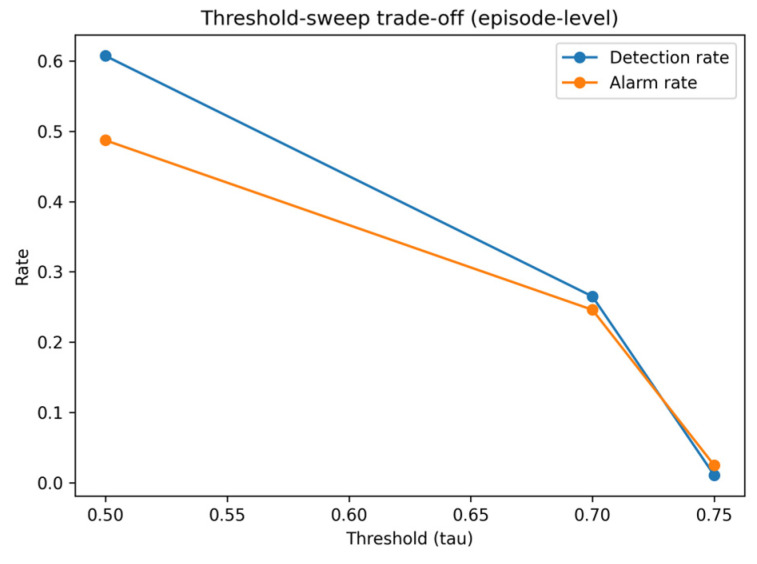
Episode-level trade-off between early-warning detection rate and alarm rate across different threshold values.

Over this evaluable subset, the threshold sweep reveals a steep trade-off between early-warning detection and alarm burden. The threshold-dependent trade-off between detection performance and alarm burden is illustrated in Figure 7. At a low threshold (τ = 0.50), the early-warning detection rate reaches 0.607, but the alarm rate increases to 0.487. This detection rate is computed at the episode level over the 1041 evaluable episodes defined above. Increasing the threshold reduces alarm volume, but detection performance decreases rapidly: at τ = 0.70, the detection rate drops to 0.265 with an alarm rate of 0.246, while at τ ≥ 0.75, the alarm rate falls below 0.025 and the detection rate decreases to 0.011. These episode-level results indicate that improved event detection can be achieved only at the cost of substantially increased alarm burden.

These results show that achieving moderate early-warning detection requires a substantially elevated alarm rate, whereas thresholds that maintain low alarm burden result in near-zero detection performance. This trade-off characterizes the limitations of a single-threshold alarm policy under the evaluated conditions. At the same time, the window-level operating points in Table 12 show that threshold selection can still be tuned to different operational objectives, such as maximizing F1-score or satisfying an alarm-rate budget. Therefore, E4 should be interpreted not as the search for a universally optimal threshold, but as an analysis of how operational priorities shape the feasible decision region.

To examine whether this limitation depends strongly on the specific aggregation rule, additional score-level comparisons were conducted using the final decision score (*prob*), the mean horizon-wise score (*tx_prob_mean*), and the raw XGBoost probability term (*xgb_prob*). In the current implementation, the final decision score ‘*prob*’ is mathematically equivalent to the maximum horizon-wise score (*tx_prob_max*) by construction, consistent with Equation (3). Accordingly, ‘*prob*’ and ‘*tx_prob_max*’ yield identical ROC-AUC and PR-AUC values, and this equality should be interpreted as an expected consequence of the score definition rather than as an independent empirical result. The results are summarized in Table 13. Relative to the max-based decision score, the mean-based score performed slightly worse (ROC-AUC = 0.6799, PR-AUC = 0.003134). In contrast, the raw XGBoost probability did not provide a usable operational score under the evaluated setting.

These additional comparisons suggest that the strong trade-off observed in E4 is not merely an artifact of the specific final score implementation. Rather, closely related aggregation rules produce qualitatively similar behavior, while weaker averaged alternatives do not materially resolve the limitation. This reinforces the interpretation that the observed difficulty is structural at the decision layer, rather than a consequence of one narrowly chosen thresholding formula.

### 5.5. Interpretability Analysis of the Proposed Framework

To improve interpretability and address reviewer concerns regarding model explanation, SHAP-based analyses were conducted at two levels: (i) a raw-feature baseline XGBoost model and (ii) the decision layer of the proposed framework. This two-level analysis was designed to distinguish feature importance in direct raw-signal classification from importance in the aggregated alarm-oriented decision process.

**Figure 8:**
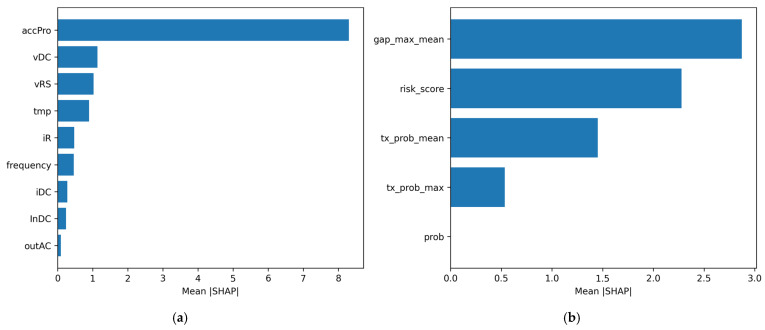
SHAP-based interpretability analysis at two levels: (**a**) sensor-level summary of mean absolute SHAP values for the raw-feature baseline XGBoost model and (**b**) decision-layer SHAP importance for the proposed framework.

For the raw-feature baseline model, SHAP results indicate that a limited subset of variables contributes disproportionately to prediction. As summarized in Figure 8a and Table 14, accumulated power generation (*accPro*) shows the strongest contribution, followed by DC voltage (*vDC*), AC line voltage between phases R and S (*vRS*), and inverter temperature (*tmp*). Additional contributions are observed from phase current (*iR*), grid frequency (*frequency*), DC current (*iDC*), and input DC current (*InDC*), whereas several other variables contribute marginally under the evaluated setting. This pattern suggests that raw-signal prediction is driven primarily by a small group of operational and electrical indicators rather than by uniform contributions across all input variables.

The SHAP analysis also indicates that raw-feature importance is temporally localized. In the baseline model, the largest contributions are concentrated at a limited number of time lags within the 56-step input window. This result suggests that the baseline decision process is influenced not only by which variables are observed, but also by when precursor patterns appear within the recent history window.

For the proposed framework, SHAP analysis of the decision layer shows a different pattern. As shown in Figure 8b and Table 14, the most influential terms are the difference between maximum and average predicted probabilities across horizons (*gap_max_mean*), the aggregated operational risk score (*risk_score*), the mean predicted probability across horizons (*tx_prob_mean*), and the maximum predicted probability across horizons (*tx_prob_max*). In contrast, no additional independent contribution was attributed to ‘*prob*’ in the decision-layer SHAP representation, because the final decision score is defined through the max-based aggregation structure and therefore overlaps with other derived decision terms. This indicates that alarm-oriented decision behavior is governed primarily by the interaction between aggregated multi-horizon risk measures and internal score differences, rather than by a single scalar probability alone.

Taken together, these results suggest that the proposed framework operates at two interpretable levels. At the raw-signal level, a limited set of operational variables dominates prediction. At the decision level, alarm generation is shaped mainly by aggregated multi-horizon risk structure rather than by a single posterior probability. This interpretation supports the view that the proposed framework should be understood not only as a predictive model, but also as a structured decision pipeline.

## 6. Discussion

This study examined multi-horizon fault prediction for photovoltaic (PV) inverters under operational constraints, with a focus on how predictive outputs translate into actionable decisions in real deployment settings. Unlike conventional approaches that focus primarily on predictive accuracy, this study explicitly analyzes how probabilistic outputs behave when converted into operational alarm decisions.

The results show that multi-horizon prediction provides meaningful early-warning information, but only within a limited temporal range. While ranking performance remains relatively stable across horizons, precision–recall performance degrades as the prediction horizon increases, indicating that usable lead time is concentrated in near-term horizons. This suggests that multi-step prediction should be interpreted as defining an effective decision horizon rather than uniformly extending predictive foresight.

Under severe class imbalance, the results further show that apparent predictive performance can be misleading when evaluated using threshold-independent metrics alone. While ROC-AUC remains high across configurations, improvements in minority-event detection are reflected primarily in precision–recall space. The comparison of imbalance-handling strategies demonstrates that robustness is not an inherent property of the model, but emerges from explicit design choices. In particular, combining multiple imbalance corrections does not necessarily yield additive benefits and can degrade performance, indicating that imbalance handling must be tuned rather than accumulated. This highlights the importance of evaluating models in regimes that reflect operational rarity rather than relying on aggregate ranking metrics.

Additional baseline experiments further emphasize the difficulty of the problem setting. Under the evaluated 23-day rare-event regime, a simpler LSTM baseline converged to near-random behavior, indicating that a conventional temporal model may fail to learn useful fault-discriminative structure under extreme event sparsity and temporally concentrated positives. Likewise, the comparison between the aggregated any-horizon target and the farthest single-horizon target (h=15) showed that the single-horizon model achieved slightly higher ROC-AUC but lower PR-AUC. This suggests that a distant single horizon can remain rank-separable while still being less informative in precision–recall terms because of its much lower event prevalence. From an operational perspective, this supports the use of an aggregated multi-horizon target, which better preserves practical decision relevance under rare-event conditions.

Temporal variation introduces an additional layer of complexity. The block-wise evaluation shows that detection performance varies substantially across time, even under a fixed model and threshold. This variability suggests that operational drift affects not only feature distributions but also the reliability of early-warning signals, and that robustness cannot be assumed from static evaluation alone. Therefore, temporal stability should be treated as a primary evaluation dimension rather than a secondary diagnostic. Moreover, the median lead times remain close to the 15 min prediction window across weekly blocks, indicating that alarms tend to occur near the end of the available lead window rather than substantially earlier. This limits the practical flexibility of intervention even when an event is successfully detected.

The most critical finding emerges from the threshold-sweep analysis. The results demonstrate that threshold-based alarm policies exhibit a structural trade-off between detection performance and alarm burden. Moderate early-warning detection can be achieved only at the cost of high alarm rates, whereas thresholds that satisfy realistic alarm constraints result in near-zero detection. This indicates that a single-threshold policy cannot simultaneously achieve both high sensitivity and low alert burden under severe class imbalance. Importantly, this limitation is not specific to the proposed model, but arises from the fundamental interaction between probabilistic prediction and binary decision rules under rare-event conditions.

Additional score-level comparisons indicate that this limitation is not fully explained by one specific aggregation choice. The final decision score and the maximum-based horizon score produced nearly identical discrimination, whereas the mean-based aggregation was only slightly weaker, and the raw classifier probability did not provide a usable operational score under the evaluated setting. This suggests that the observed trade-off is not merely an artifact of one narrowly chosen score formulation. Instead, closely related aggregation rules tend to preserve the same qualitative limitation, implying that more flexible alternatives such as decay-weighted aggregation, persistence-based logic, or horizon-specific decision rules may be needed to improve practical usability.

Most existing studies on fault prediction primarily evaluate model performance using metrics such as accuracy or AUC, implicitly assuming that improved predictive performance translates into improved operational decisions. However, the results of this study show that this assumption does not hold in severely imbalanced and decision-constrained environments. Even when predictive performance appears strong, the resulting alarm behavior can be fundamentally limited by thresholding effects and class imbalance. This gap between predictive performance and decision utility suggests that model evaluation should explicitly incorporate decision-level behavior. In this sense, prior work on false-alarm control and decision-oriented monitoring is relevant, but the present results show that the problem becomes especially acute when multi-horizon PV inverter predictions must be translated into operational alarms under extreme event sparsity.

This indicates that the key challenge in operational fault prediction is not only to improve predictive accuracy, but to ensure that prediction outputs can be translated into usable decisions under realistic constraints. In this context, the limitation observed in threshold-based alarm policies is not a parameter-tuning issue, but a structural characteristic of the decision process itself. Accordingly, system design must consider not only how predictions are generated, but also how they are aggregated, calibrated, and operationalized.

Taken together, these findings suggest that operational fault prediction is fundamentally constrained by the interaction between prediction uncertainty, class imbalance, temporal variation, and decision policy design. Improving predictive accuracy alone is insufficient to ensure practical usability; instead, system-level design must account for how predictions are aggregated, calibrated, and converted into decisions under operational constraints. This perspective shifts the focus from model-centric optimization to decision-aware system design.

Several limitations remain. The temporal coverage of the evaluation is limited, restricting the ability to fully characterize long-term drift and seasonal effects. In addition, the current alarm policy is based on a single-threshold mechanism, which may not capture more complex operational requirements such as persistence, cost asymmetry, or asset-specific priorities. Furthermore, the interpretability analysis is based on aggregated SHAP summaries, and more detailed investigation of temporal feature interactions could provide additional insights. The additional baseline comparisons were also performed on positive-containing intervals because positives are strongly concentrated within limited parts of the 23-day record, which itself reflects the difficulty of evaluation under real operational rarity.

Future work should therefore focus on extending temporal evaluation, exploring alternative aggregation and calibration strategies, and developing decision policies that move beyond single-threshold formulations to better reflect real operational environments. In particular, approaches that integrate adaptive thresholding, cost-sensitive decision rules, persistence-based logic, or sequential alarm policies may provide more practical solutions under real-world constraints.

## 7. Conclusions

This study presented an operation-aware framework for multi-horizon fault prediction in photovoltaic (PV) inverters, integrating TimeXer-based temporal representation learning with probabilistic classification and threshold-based alarm generation under real-time constraints.

The results show that multi-horizon prediction can provide meaningful early-warning signals, but only within a limited lead-time range where precision–recall performance remains informative. Under severe class imbalance, imbalance-aware training substantially improves minority-event detection, although performance remains sensitive to temporal variation. Additional baseline experiments further showed that a simpler temporal model can collapse under the evaluated rare-event setting, while the aggregated multi-horizon target remains more informative in precision–recall terms than a distant single-horizon target. Most importantly, the threshold-sweep analysis reveals that alarm generation is fundamentally constrained by a trade-off between detection performance and alert burden. The interpretability analysis further shows that the proposed framework is governed not only by raw operational variables, but also by aggregated multi-horizon decision terms that shape alarm behavior.

These findings indicate that the primary challenge in operational fault prediction is not only to improve predictive accuracy, but to ensure that predictive outputs can be translated into reliable decisions under real-world constraints. In particular, the observed limitations of single-threshold alarm policies highlight that decision-level behavior must be considered explicitly when designing predictive maintenance systems. Additional score-level comparisons also suggest that this limitation is not fully resolved by simply substituting closely related aggregation rules. Accordingly, the main contribution of this study lies not only in predictive modeling, but also in showing that decision-aware evaluation is essential for operational PV fault forecasting.

Future work should extend evaluation to longer temporal spans, explore alternative aggregation and calibration strategies for multi-horizon outputs, and develop decision policies that better reflect operational constraints such as cost, asset criticality, and alarm persistence. Additional work is also needed to investigate adaptive thresholding and more flexible alarm policies that can better balance early detection and alert burden under rare-event conditions.

## Figures and Tables

**Table 1:** Overall prevalence of fault-positive windows in the labeled dataset.

Item	Count	Share (%)
Total labeled windows	749,304	100.000
Fault-positive windows	1402	0.187
Fault-negative windows	747,902	99.813

**Table 2:** Broad fault event categories in the operational event log and their prevalence relative to the labeled dataset.

Broad Fault Category	Event-Log Count	Share of All Labeled Windows (%)	Share of Event-Log Labels (%)	Used as a Separate Class
Electrical	4924	0.657	62.5	No
Thermal	1422	0.190	18.1	No
Grid-interaction	1410	0.188	17.9	No
Control/protection	122	0.016	1.5	No
Total event-log labels	7878	1.051	100.0	

**Table 3:** Hardware/software environment used for the real-time deployability test (E1).

Category	Specification
Operating system	Microsoft Windows 11 Home (Version 10.0.26100, Build 26100)
Processor	12th Gen Intel(R) Core(TM) i9-12900K @ 3.187 GHz
CPU configuration	16 cores, 24 logical processors
System type	x64-based PC
Installed RAM	128 GB
Motherboard	ASRock H670 PG Riptide
BIOS	American Megatrends International, LLC (AMI), Norcross, GA, USA; 4.01 (6 December 2021)
Inference setting	CPU-only sequential streaming evaluation
Evaluated streams	24 inverter streams (unique plant–equipment pairs)
Warm-up exclusion	First 200 windows per stream
Maximum evaluated windows	Up to 5000 windows per stream
Sampling interval	1 min

**Table 4:** Real-time inference latency and throughput summary across 24 inverter streams.

Metric	Median	Min	Max
Throughput (windows/s)	585.287	446.997	628.226
Mean latency (ms)	1.104	1.008	1.589
P95 latency (ms)	1.295	1.157	1.636
P99 latency (ms)	1.473	1.282	2.889
Preprocessing (ms)	0.077	0.073	0.085
Embedding (ms)	0.584	0.534	0.856
XGBoost (ms)	0.433	0.391	0.636
Post-processing (ms)	0.008	0.008	0.009

**Table 5:** Multi-horizon lead-time prediction performance at representative forecast steps with horizon-wise positive rates.

Horizon (*h*)	Positive Rate	ROC-AUC	PR-AUC
1	0.0017	0.8138	0.0828
3	0.0017	0.7712	0.0276
5	0.0017	0.8286	0.0368
10	0.0018	0.7708	0.0281
15	0.0019	0.7563	0.0250

**Table 6:** Results for imbalance-handling strategies on the offline labeled window dataset reported using ROC-AUC and PR-AUC.

Model	ROC-AUC	PR-AUC	Samples	Positive Rate
XGBoost baseline (no imbalance handling)	0.9096	0.3665	100,667	0.0258
XGBoost with class weighting	0.8989	0.5539		
XGBoost with training-only oversampling	0.9061	0.5579		
XGBoost with class weighting and oversampling	0.8973	0.5415		

**Table 7:** Common-tail fixed-cutoff comparison at τ = 0.5 between the raw baseline score and the proposed decision score under matched evaluation coverage.

Model	Samples	F1 at 0.5	Alarm Rate at 0.5
Raw XGBoost baseline	299,550	0.0032	0.0021
Proposed hybrid	299,550	0.0001	0.1931

**Table 8:** Bootstrap-based confidence interval summary for representative ROC-AUC and PR-AUC estimates.

Model	n	Positives	ROC-AUC	95% CI of ROC-AUC	PR-AUC	95% CI of PR-AUC
Baseline	655,080	47	0.9371	0.9244–0.9500	0.00918	0.00299–0.01960
Proposed	346,565	736	0.6863	0.6757–0.6958	0.00318	0.00294–0.00346

**Table 9:** Additional simpler temporal baseline under the rare-event setting.

Model	Evaluation Subset	Positive Rate (Test)	ROC-AUC	PR-AUC	Note
LSTM baseline	Positive-containing interval	0.00107	0.5000	0.00107	Near-random/collapsed

**Table 10:** Single-horizon versus any-horizon comparison on the positive-containing interval.

Comparison	n	Positive Rate (Test)	ROC-AUC	PR-AUC
Any-horizon	30,000	0.00107	0.9630	0.4239
Single-horizon (h = 15)	30,000	0.00010	0.9753	0.3342

**Table 11:** Block-wise early-warning detection performance over the 23-day period.

Week	Episodes	With Stream	Detected	Detection Rate (%)	Median Lead Time (Min)	Lead Time IQR (Min)
W1	222	222	55	24.8	14.59	14.52–14.77
W2	355	310	223	71.9	14.49	14.29–14.74
W3	250	250	201	80.4	14.61	14.14–14.80
W4	369	259	154	59.5	14.35	14.09–14.80

**Table 12:** Threshold-sweep operating points under an alarm-rate budget (window-level test performance).

Policy	Threshold	Precision	Recall	F1	Alarm Rate	Alpha
Best F1	0.63	0.839	0.693	0.759	0.021	-
Under Alpha	0.65	0.893	0.438	0.588	0.012	0.02
Closest to Alpha	0.64	0.854	0.671	0.751	0.020	0.02

**Table 13:** Comparison of operational decision scores under the evaluated aggregation setting.

Score	n	Positives	ROC-AUC	PR-AUC
*prob*	346,565	736	0.6863	0.003182
*tx_prob_mean*	346,565	736	0.6799	0.003134
*xgb_prob*	0	0	–	–

**Table 14:** SHAP summary for the raw-feature baseline model and the proposed decision layer.

Analysis Level	Variable/Term	Interpretation	Mean Absolute SHAP
Raw-feature baseline	*accPro*	Cumulative power generation (operational production state)	8.3007
Raw-feature baseline	*vDC*	DC-side voltage condition	1.1343
Raw-feature baseline	*vRS*	AC line-to-line voltage (R–S phases)	1.0252
Raw-feature baseline	*tmp*	Inverter thermal condition	0.8973
Raw-feature baseline	*iR*	Phase current (R phase)	0.4710
Raw-feature baseline	*frequency*	Grid frequency condition	0.4601
Raw-feature baseline	*iDC*	DC-side current	0.2729
Raw-feature baseline	*InDC*	Input DC current	0.2370
Raw-feature baseline	*outAC*	AC output power	0.0946
Decision layer	*gap_max_mean*	Difference between maximum and mean predicted probabilities across horizons	2.8732
Decision layer	*risk_score*	Aggregated operational risk score	2.2779
Decision layer	*tx_prob_mean*	Mean predicted probability across horizons	1.4530
Decision layer	*tx_prob_max*	Maximum predicted probability across horizons	0.5355
Decision layer	*prob*	Single scalar probability term	0.0000

## Data Availability

The data used in this study are derived from operational photovoltaic inverter systems and are not publicly available due to confidentiality and commercial restrictions. A subset of processed data or aggregated results may be made available by the authors upon reasonable request. The implementation details necessary to reproduce the experimental setup are described in the manuscript.

## Abbreviations

The following abbreviations are used in this manuscript:

PV	Photovoltaic
DC	Direct Current
AC	Alternating Current
ROI	Return on Investment
O&M	Operation and Maintenance
AUC	Area under the Curve
ROC	Receiver Operating Characteristic
PR	Precision-Recall
PR-AUC	Area under the Precision-Recall Curve
LSTM	Long Short-Term Memory
TCN	Temporal Convolutional Network
XGBoost	Extreme Gradient Boosting
SMOTE	Synthetic Minority Over-sampling Technique
RQ	Research Question
E	Experiment
CPU	Central Processing Unit
